# Editorial: Habitability Beyond Earth

**DOI:** 10.3389/fmicb.2018.02645

**Published:** 2018-11-15

**Authors:** Karen Olsson-Francis, Daniela Billi, Andreas Teske, Jean-Pierre P. de Vera

**Affiliations:** ^1^School of Environment, Earth and Ecosystem Sciences, The Open University, Milton Keynes, United Kingdom; ^2^Deparment of Biology, University of Rome Tor Vergata, Rome, Italy; ^3^Department of Marine Sciences, University of North Carolina at Chapel Hill, Chapel Hill, NC, United States; ^4^Astrobiological Laboratories, German Aerospace Center (DLR), Institute of Planetary Research, Management and Infrastructure, Berlin, Germany

**Keywords:** astrobiology, space, habitability, mars, extremophiles, analog sites

The question of whether Earth is a unique location for life remains one of the most enduring questions of our time. Geochemical data suggests that habitable environments may exist, or may have existed, elsewhere in the Solar System with promising targets including Mars and icy bodies where liquid water is believed to exist (Kargel, 2000; Grotzinger et al., 2014; Glein et al., 2015). Furthermore, potential habitable Exoplanets have been discovered where potentially there is sufficient atmospheric pressure to maintain liquid water (Jenkins et al., 2015; Gillon et al., 2017; Orosei et al., 2018). Yet, for life to exist it is not solely dependent on liquid water as it also needs bio-essential elements, an energy source, and the environmental conditions, that are conducive to life (Nixon et al., 2013). To investigate the feasibility of life elsewhere in the Solar System a combination of field and laboratory based studies, *in-situ* space experiments, and theoretical modeling is required. Here, we present 14 original research papers, one mini review, and two hypothesis and theory papers highlighting the novel and diverse methods that are employed to investigate potential life beyond the Earth. The overall focus of this collection of work is to understand if terrestrial life could exist elsewhere in the Solar System, and if so, what evidence (bio-signatures) could be used to support or negate the hypothesis of life.

Our understanding of life in extreme environments on Earth forms the basic concepts of where life could exist elsewhere in the Solar System. Extremophilic microorganisms have adapted to live in environments where parameters, such as, pH, temperature and pressure, and water availability are deemed extreme. Determining the limits of life in regard to these parameters is important for defining the limits of life. As Schulze-Makuch et al. demonstrates the limits of terrestrial life can be used to outline a range of possible habitable environments, some that are present in our Solar System and others that are hypothetical.

Extreme environments on Earth can also be used as terrestrial analog sites. These are sites that exhibit similar environmental conditions, such as pH, pressure, atmosphere composition, and water availability, as environments on other planets or moons (Martins et al., 2017). Historically analog studies have predominantly been focused on Mars. Evidence proposes that conditions on early Mars were clement and less oxidizing than they are today (e.g., Carr and Head, 2010; Mangold et al., 2012). Data from Mars Science Laboratory (MSL) shows that habitable environments may have existed at Gale Crater (Grotzinger et al., 2014). Chemolithotrophy has been recommended ed as a plausible metabolism for life on Mars and using data from MSL, Price et al. suggests the feasibility of iron oxidation-nitrate reduction as a plausible metabolism for life on ancient Mars.

As the conditions on Mars evolved from wet to dry during the Hesperian period ephemeral lakes are thought to have formed. For example, the presence of hydrated magnesium sulfates within the rim of Columbia Crater is ascribed to the existence of a paleolake, which at times must have been hypersaline in nature (Wray et al., 2011). Pontefract et al. shows, using a sulfate rich analog site for the ancient hypersaline palolakes, Spotted Lake (British Columbia, Canada) that sulfate salt deposits may have offered periodically habitable environments, and could have concentrated and preserved organic materials or their biomarkers over geologic time.

On modern day Mars, the evaporitic past is evident by the widespread deposition of sulfate, perchlorates and chloride salts observed today on the martian surface (Wanke et al., 2001; Clark et al., 2005). It has been hypothesized that perchlorates may bind water from the atmosphere forming brines, which remain liquid at low temperatures (e.g., Toner and Catling, 2016). Beblo-Vranesevic et al. demonstrates that *Hydrogenothermus marinus*, a desiccation tolerant bacterium, was able to tolerate high concentrations of perchlorates, which highlights the possibility of using this microorganism as a model microorganism in future experiments. Evaporitic deposits on the surface of Mars also suggests that water in the near sub-surface would by saline. Recent work has emphasized that the hypersaline springs on Axel Heiberg are a unique analog to represent putative subsurface aquifers on Mars (e.g., Sapers et al.). Based on the microbial diversity within these hypersaline springs, Sapers et al. shows that even a small chemical variation in propinquities sites in the martian sub-surface would have significant implications for community structure, and resulting bio-signatures.

Increasingly, data suggests that habitable environments may exist in the sub-surface oceans of the icy moons. For example, the Galileo, Cassini-Huygens, and Hubble Space Telescope missions support the theory of a potential briny ocean beneath the outer ice shells of Europa, Ganymede and Enceladus. Using environmental characteristics of icy worlds and terrestrial glaciers and ice sheets, Garcia-Lopez et al. concludes that the icy worlds such as Europa and Enceladus are the most likely locations to harbor life of the Solar System.

Analogue environments can also be used to test and develop new instrumentation for future life detection missions. Ideally these methods are low cost with small mass and energy requirement. Using the Canadian high Artic as an analog, Goordial proposes three techniques: the cryo-iPlate for culturing microorganisms (2) a Microbial Activity Microassay (MAM) plate (BIOLOG Ecoplate) for detecting viable extant microorganisms, and (3) the Oxford Nanopore MinION for nucleic acid detection and sequencing. Additionally, based on work carried out in the hyper-arid Namib Desert, Hinchliffe et al. recommends advanced photogrammetry as a method for future autonomous rovers to detect viable surface colonization on the surface of Mars. However, as Fox and Strasdeit discusses there are problems with misinterpreting bio-signatures on other planets and moons that need to be considered.

In addition to environmental analog studies, laboratory simulation experiments are used to further our understanding of potential processes on Mars. Based on data from past mission, Mars regolith analog material can be prepared and used to study potential biogeochemical cycling on Mars. Using Mars simulants as a source of metals, Kölbl et al. demonstrates that surface bioprocesses on the regolith surface could be used as a bio-signature for future missions. However, laboratory based experiments are short-term and Olsson-Francis et al. shows that combining laboratory based experiments with thermochemical modeling is a feasible method for identifying geochemical bio-signatures that are produced over geological timescales. Laboratory based simulation experiments are also used to determine the effect of the extraterrestrial conditions on microbial survivability and activity. For example, de la Torre Noetzel et al. establishes that lichens can survive 30 days in simulated Mars conditions, but the photobiont was unable to perform photosynthesis under these conditions. Microorganisms have been extensively studied under simulated conditions at the surface of Mars (for review see Olsson-Francis and Cockell, 2010). However, Bak et al. proves for the first time that stress effect induced by silicates abraded in a Mars-like atmosphere would be detrimental to life at the martian surface.

Laboratory simulation experiments have revealed that ionizing radiations represents the major hazard for microbial survival, persistence of detectable biosignatures, and operation of spacecraft equipment (Dartnell, 2011). The international STARLIFE-irradiation campaign studied the response of increased doses of ionizing radiation and heavy ions, mimicking Galactic cosmic rays, on astrobiological relevant microorganisms (Moeller et al., 2017). As part of this study, Pacelli et al. illustrates that exposure of the black fungus *Cryomyces antarcticus* CCFEE 515 showed that the fungus maintained high survival and metabolic activity with no detectable DNA and ultrastructural damage, even after the highest dose irradiation.

However, to fully understand the effect of extraterrestrial conditions on microorganisms and bio-signatures a combination of laboratory based and *in-situ* space experiments are required. Exposure experiments in Low Earth Orbit (LEO) exposes samples to several radiation types, such as ionizing, UV, and cosmic radiation (galactic cosmic rays and solar particle events) combined with other conditions, such as vacuum and dust bombardment, which cannot be simulated on Earth. Long-term exposure experiments are carried out on the outside on the International Space Station (ISS). Exposure facilities include the ESA funded EXPOSE-R and an in-depth description of the facility is described in detail by Rabbowet et al.. The samples are exposed long-term to the conditions of LEO to investigate the effect of exposure on microorganisms and their associated bio-signatures, before returning to Earth for analysis (for review see Cottin et al., 2017). Bio-signatures include biomarkers, such as carotenoid deinixanthin, which can be used as evidence on past life on Mars. Leuko et al. demonstrates that this biomarker is strongly resistant to LEO conditions and simulated Mars conditions (when protected from solar radiation), suggesting that it could be used as a target for future missions.

In future, as technology develops microorganisms could play a key part in space exploration, such as *in-situ* resource utilization, and life support systems. On Earth, previous work has shown that the microorganism C. *metallidurans* CH34 is able to leach bio-essential elements from basaltic material (Olsson-Francis et al., 2010). Building on this work, Byloos et al. investigates the effect of space flight and long-term storage on *C. metallidurans* CH34 and interactions with basaltic material (a lunar-type rock), which was the first step to determining the feasibility of bio-mining in space. Although more work is needed the results may “open the door future studies and potential application in space.”

With future missions planned to Mars and the icy moons, understanding the limits of microbial life and their associated bio-signatures is vital. This Research Topic presents advances in our understanding of habitability and bio-signatures using a suit of state-of-the-art methods. To date research has predominantly focused on Mars, but as our understanding of the icy moons increases, our attention most expands to the outer Solar System.

## Author contributions

All authors listed have made a substantial, direct and intellectual contribution to the work, and approved it for publication.

### Conflict of interest statement

The authors declare that the research was conducted in the absence of any commercial or financial relationships that could be construed as a potential conflict of interest.

## References

[B1] Carr M. H. Head J. W. (2010). Geologic history of Mars. Earth Planet. Sci. Lett. 294, 185–203. 10.1016/j.epsl.2009.06.042

[B2] Clark B. C. Morris R. V. Mclennan S. M. Gellert R. Jolliff B. Knoll A. H. (2005). Chemistry and mineralogy of outcrops at Meridiani Planum. Earth Planet. Sci. Lett. 240, 73–94. 10.1016/j.epsl.2005.09.040

[B3] Cottin H. Kotler J. M. Billi D. Cockell C. Demets R. Ehrenfreund P. (2017). Space as a tool for astrobiology: review and recommendations for experimentations in earth orbit and beyond. Space Sci. Rev. 209, 83–181. 10.1007/s11214-017-0365-5

[B4] Dartnell L. R. (2011). Ionizing radiation and life. Astrobiology 11, 551–582. 10.1089/ast.2010.0528 21774684

[B5] Gillon M. Triaud A. H. M. J. Demory B.-O. Jehin E. Agol E. Deck K. M.. (2017). Seven temperate terrestrial planets around the nearby ultracool dwarf star TRAPPIST-1. Nature 542, 456–460. 10.1038/nature21360 28230125 PMC5330437

[B6] Glein C. R. Baross J. A. Waite J. H. (2015). The pH of Enceladus' ocean. Geochim. Cosmochim. Acta 162, 202–219. 10.1016/j.gca.2015.04.017

[B7] Grotzinger J. P. Sumner D. Y. Kah L. C. Stack K. Gupta S. Edgar L.. (2014). A.T.M.T. (2014). A habitable fluvio-lacustrine environment at Yellowknife Bay, Gale Crater, Mars. Science 343:14. 10.1126/science.1242777 24324272

[B8] Jenkins J. M. Twicken J. D. Batalha N. M. Caldwell D. A. Cochran W. D. Endl M. (2015). Discovery and validation of Kepler-452b: A 1.6 R_⊕_ Super Earth Exoplanet in the habitable zone of a G2 Star. Astronom. J. 150:56 10.1088/0004-6256/150/2/56

[B9] Kargel J. S. E. A. (2000). Europa's crust and ocean: origin, composition, and the prospects for life. Icarus 148, 226–265. 10.1006/icar.2000.6471

[B10] Mangold N. Kite E. S. Kleinhans M. G. Newsom H. Ansan V. Hauber E. (2012). The origin and timing of fluvial activity at Eberswalde crater, Mars. Icarus 220, 530–551. 10.1016/j.icarus.2012.05.026

[B11] Martins Z. Cottin H. Kotler J. M. Carrasco N. Cockell C. S. Noetzel R. D. (2017). Earth as a tool for astrobiology-a european perspective. Space Sci. Rev. 209, 43–81. 10.1007/s11214-017-0369-1

[B12] Moeller R. Raguse M. Leuko S. Berger T. Hellweg C. E. Fujimori A.. (2017). STARLIFE-an international campaign to study the role of galactic cosmic radiation in astrobiological model systems. Astrobiology 17, 101–109. 10.1089/ast.2016.1571 28151691

[B13] Nixon S. L. Cockell C. S. Cousins C. R. (2013). Plausible microbial metabolisms on Mars. Astronomy Geophys. 54, 13–16. 10.1093/astrogeo/ats034

[B14] Olsson-Francis K. Cockell C. S. (2010). Experimental methods for studying microbial survival in extraterrestrial environments. J. Microbiol. Methods 80, 1–13. 10.1016/j.mimet.2009.10.004 19854226

[B15] Olsson-Francis K. Van Houdt R. Mergeay M. Leys N. Cockell C. S. (2010). Microarray analysis of a microbe-mineral interaction. Geobiology 8, 446–456. 10.1111/j.1472-4669.2010.00253.x 20718869

[B16] Orosei R. Lauro S. E. Pettinelli E. Cicchetti A. Coradini M. Cosciotti B.. (2018). Radar evidence of subglacial liquid water on Mars. Science 361, 490–493. 10.1126/science.aar7268 30045881

[B17] Toner J. D. Catling D. C. (2016). Water activities of NaClO_4_, Ca(ClO_4_)_2_, and Mg(ClO_4_)_2_ brines from experimental heat capacities: Water activity > 0.6 below 200 K. Geochim. Cosmochim. Acta 181, 164–174. 10.1016/j.gca.2016.03.005

[B18] Wanke H. Bruckner J. Dreibus G. Rieder R. Ryabchikov I. (2001). Chemical composition of rocks and soils at the Pathfinder site. Space Sci. Rev. 96, 317–330. 10.1023/A:1011961725645

[B19] Wray J. J. Milliken R. E. Dundas C. M. Swayze G. A. Andrews-Hanna J. C. Baldridge A. M. (2011). Columbus crater and other possible groundwater-fed paleolakes of Terra Sirenum, Mars. J. Geophys. Res. Planets 116:3694 10.1029/2010JE003694

